# The role of headache chronicity among predictors contributing to quality of life in patients with migraine: a hospital-based study

**DOI:** 10.1186/1129-2377-15-68

**Published:** 2014-10-02

**Authors:** Sun-Young Kim, Sung-Pa Park

**Affiliations:** 1 Department of Neurology, University of Ulsan College of Medicine, Ulsan University Hospital, 290-3 Jeonha-dong, Dong-gu, Ulsan 682-714, Republic of Korea; 2 Department of Neurology, School of Medicine, Kyungpook National University, 680 Gukchaebosang-ro, Jung-gu, Daegu 700-842, Republic of Korea

**Keywords:** Chronic migraine, Headache, Disability, Depression, Anxiety, Quality of life

## Abstract

**Background:**

Headache chronicity has been known to elicit deleterious effects on quality of life (QOL). We evaluated the contribution of headache chronicity to QOL in relation to clinical, psychiatric, and psychosocial variables in patients with migraine.

**Methods:**

Subjects were recruited from a headache clinic and completed self-report questionnaires including the Migraine Disability Assessment (MIDAS), Beck Depression Inventory (BDI), Beck Anxiety Inventory (BAI), and Migraine-Specific Quality of Life (MSQoL). We obtained predictors of MSQoL by multiple regression analyses. A path analysis model was constructed to analyze interrelationships between the variables.

**Results:**

Among 251 eligible patients, 183 (72.9%) had episodic migraine (EM) and 68 (27.1%) had chronic migraine (CM). Patients with CM had more serious clinical, psychiatric, and poor QOL than did patients with EM. The strongest predictor of the MSQoL score in all patients with migraine was the BDI score (β = -0.373, *p* < 0.001), followed by the MIDAS score (β = -0.223, *p* < 0.001), female gender (β = -0.192, *p* < 0.001), attack duration (β = -0.159, *p* = 0.001), and headache chronicity (β = -0.130, *p* = 0.012). Headache chronicity had a direct effect on the MSQoL score and exerted an indirect effect on the MSQoL score through the MIDAS and the BDI scores.

**Conclusions:**

Chronic migraine appears to impair QOL directly as well as indirectly by provoking disability and depression.

## Article highlights

•Patients with chronic migraine were more likely to have a lower education level, higher attack frequency, higher pain intensity, and higher frequency of cephalic allodynia, than those with episodic migraine.

•QOL in patients with chronic migraine is also significantly lower, because of both the direct effects of chronicity, and the indirect (increasing the frequency of disability and depression) effects.

## Background

In the newly released version of the Global Burden of Disease study of 2010, migraine is responsible for almost 3% of disability attributable to a specific disease worldwide. After including its comorbidities, migraine should be considered among the most disabling of diseases [1]. Indeed, it is considered the most disabling among neurological disorders, and the eighth most burdensome disease [1,2]. Migraine is a common neurological disorder, with 1-year prevalence ranging from 4.5% to 8% for men, and from 8% to 16% for women [3-5]. In a population-based survey from Korea, the prevalence of migraine is somewhat lower, at 2.9% for men and 9.2% for women [6]. Migraine can be considered a chronic disorder, with episodic attacks and potential progression to more frequent and severe attack patterns [7]. With regard to headache chronicity, migraine is classified into episodic migraine (EM) and chronic migraine (CM). CM is defined by the International Classification of Headache Disorders, second edition (ICHD-2), as a headache frequency of ≥15 days per month on average over the preceding 3 months [8]. Although the prevalence of CM in the general population is as low as 1.4%–2.2% [9], the disease burden of CM was highlighted in several studies [10-13]. In the American Migraine Prevalence and Prevention (AMPP) study, patients with CM had 2 times higher developing depression, anxiety, and chronic pain than those with EM [14]. In addition, health care costs and productivity loss at work were larger in patients with CM than those with EM [10,11]. The International Burden of Migraine Study (IBMS) collected data from several countries in Western Europe, North America and the Asia/Pacific regions, and reported that patients with CM demonstrated greater disability, a lower quality of life (QOL), and higher levels of anxiety and depression, than those with EM [12,13]. CM and depression are in fact known risk factors for suicide behavior or ideation in patients with migraine [15,16].

Reduced QOL was reported in patients with migraine when compared with age- and sex-matched people without migraine [17-19]. Patients with migraine demonstrated significant impairment of QOL compared to the controls, independent of depression or anxiety [20,21]. Although several predictors for QOL in patients with migraine were proposed, depression and migraine-associated factors were the most consistent ones [12,14,17,20,22-24]. Migraine disability [20,22,24], headache intensity [22-24], CM [12,14,19], and attack frequency [20,23,24] were involved in migraine-associated factors. A community-based study for women with migraine analyzed the interrelationship of headache intensity, chronic pain experience, and depression with disability or QOL, using the regression model [23]. However, it included only women as a study population and did not consider CM as a risk factor. The purposes of the study are 1) to examine clinical, psychiatric, and QOL of CM compared to EM; and 2) to evaluate how headache chronicity affects QOL in relation to other variables, including depression and anxiety.

## Methods

### Subjects

We included patients with migraine who attended the outpatient clinic in Department of Neurology at Kyungpook National University Hospital from November 9, 2010 to August 31, 2012. A diagnosis of migraine was assigned based on the International Classification of Headache Disorders, second edition (ICHD-2) [8]. Headache chronicity was defined as whether patients had EM or CM. The definition of CM was determined by the criteria of ICHD-2 Revised, which considered CM as ≥15 headache days per month on average over the preceding 3 months, and with at least 8 days/month either meeting criteria for migraine without aura or responding to migraine-specific medication [25]. This study included patients who were between 15 and 70 years of age and who agreed to participate in the study. Patients who took migraine-specific medications (triptans, ergot, or preventive medicines) or psychotropic agents (antidepressants, anxiolytics, or antipsychotics) at any time in the preceding year were excluded. Patients with severe medical, psychiatric, or neurological disorders, mental retardation, and alcohol or drug abuse that prevented them from understanding the questionnaires and cooperating with the study were also excluded.

### Study design

This is a cross-sectional study and each patient was enrolled consecutively. Each patient was interviewed by a trained neurologist (SPP) who also reviewed the patient’s medical charts to collect demographic and clinical information for a computerized database. Sociodemographic data were collected on variables including age, gender, education, job (having a job or not), income (earning at least 1 million KRW per month, equivalent to 980 USD per month or not), marital status (married or single, divorced, and bereaved), and concurrent medical diseases (suffering from medical diseases or not). Clinical data included age at onset, disease duration, attack frequency and duration, headache intensity, accompanying symptoms (e.g., photophobia, phonophobia, and cephalic allodynia) and family history. Attack frequency meant the number of migraine attacks for preceding 3 months. Headache intensity was measured by the Visual Analog Scale (VAS). The VAS was measured in two different ways—the VAS_max_ and VAS_now_. VAS_max_ meant the maximal intensity of headaches experienced during the prior 3 months, and VAS_now_ represented the intensity of headache on the day of answering the self-report questionnaires. Cephalic allodynia was assessed by a simple questionnaire with dichotomous options (yes or no). Patients were asked to recall whether they had abnormal skin sensitivity on their face or scalp especially when they engaged in any of the following activities during migraine attack: (1) combing hair; (2) pulling hair back; (3) shaving face; (4) wearing eyeglasses; (5) wearing earrings; (6) taking a shower (when shower water hits the face); (7) being exposed to cold. If patients experienced any of these, we considered them positive for allodynia. Family history of migraine was defined as the existence of migraine in a lineal ascendant and siblings. All subjects completed reliable and validated self-report questionnaires, including the Korean versions of the Migraine Disability Assessment Scale (MIDAS) [26], the Beck Depression Inventory (BDI) [27], the Beck Anxiety Inventory (BAI) [28], and the Migraine-Specific Quality of Life (MSQoL) [29]. We compared clinical, psychiatric, and QOL of CM with those of EM, and evaluated the role of CM on QOL with respect to other variables.

### Questionnaires

#### Migraine Disability Assessment Scale (MIDAS)

The Korean version of the MIDAS, a five-item questionnaire designed to evaluate disability within the most recent 3 months, was used in this study [26]. Patients were asked to report decreased performance in the domains of work/school, household work, and family/social activities. Scores (0–27), measured the overall level of disability: Grade I (0–5), Grade II (6–10), Grade III (11–20), and Grade IV (above 21). Cronbach’s α value was 0.75.

#### Beck Depression Inventory (BDI)

The BDI is the most commonly used self-rating scale for depression [27]. Participants rated 21 items on a scale from 0 to 3 according to how they felt at the time. Subjects who scored above 16 were considered to have depression. Cronbach’s α value was 0.8.

#### Beck Anxiety Inventory (BAI)

The BAI is a 21-item self-report measure of anxiety severity, with each item describing a common symptom of anxiety [28]. The respondent is asked to rate how much he or she has been bothered by anxiety symptoms during the previous week on a 4-point scale (0–3). Subjects who score above 21 are considered to suffer from anxiety. Cronbach’s α values was 0.9.

#### Migraine-Specific Quality of Life (MSQoL)

The MSQoL was developed by Wagner et al., and has proven to be a valid and reliable tool in clinical migraine research [30]. It is a 25-item questionnaire translated into and validated for the Korean language [29]. The items are rated on a 4-point scale (1–4). A lower total score (overall range of 25–100) indicates poorer QOL. Cronbach’s α value was 0.93.

### Statistical analysis

Data for continuous variables were expressed as means ± SD, and those for categorized variables were expressed as frequencies. The independent *t* test, Mann-Whitney *U* test, and Fisher’s exact test were applied to compare variables between EM and CM. Not only demographic, socioeconomic, and clinical variables, but also questionnaire scores were included as independent variables to measure predictors of the MSQoL score, by multiple linear regression analyses with stepwise selection. The probabilities of entry and exit were 0.05 and 0.1, respectively. Collinearity was addressed by performing the appropriate statistics. Variables selected from the linear regression analyses were used to construct a structural equation model to test the interrelations between variables and the MSQoL score. Based on a review of previous studies [12,14,17,19,20,22-24], we developed a hypothetical model outlining the path of depression, migraine disability, gender, attack duration, headache chronicity (indicating CM), and gender to QOL. We hypothesized that all of these variables influenced on QOL directly, and gender, headache chronicity, and attack duration influenced on QOL indirectly through the mediation of migraine disability and depression. The hypothesized path model was tested with structural equation model. Model fit was evaluated using path analysis, a method that allows for estimation of the relative importance of different paths of the independent variables onto the dependent variables. An acceptable model fit was defined as having a non-significant chi-square (χ^*2*^) value, a Normed Fit Index (NFI) of ≥0.9, a Comparative Fit Index (CFI) of ≥0.9, a Goodness of Fit Index (GFI) of ≥0.9, and a Root Mean-square Residual (RMR) of ≤0.05. Structural equation modeling was used to estimate the total effect of each predictor, in order to establish a linear model to predict the MSQoL score with these interrelations accounted for. Except for the structural equation model, all statistical analyses were conducted with SPSS Version 20 (IBM SPSS Inc). LISREL 8.8 for Windows (Scientific Software International, USA) was used for the path and structural equation modeling components of the analysis. The level of statistical significance was set at 0.05.

### Ethics or Institutional review board approval

This cross-sectional study was approved by the Institutional Review Board of Kyungpook National University Hospital, and all participants provided written informed consent prior to their participation.

## Results

A total of 307 patients with migraine consecutively visited our clinic during the study period. Among them, 56 patients were excluded because of refusal to complete the questionnaires (*n* = 28); probable migraine (*n* = 12); mental retardation (*n* = 3); illiteracy (*n* = 5); or age older than 70 (*n* = 8). Subsequently, 251 patients (212 men/39 women, mean age 38.9 ± 12.9 years old) completed the study. With respect to headache chronicity, 183 patients (72.9%) had EM and 68 patients (27.1%) had CM. Demographic and clinical characteristics between EM and CM are documented in Table 1. Patients with CM had a lower level of education (*p* = 0.002), higher attack frequency (*p* < 0.001), higher VAS_now_ score (*p* < 0.001), and higher frequency of cephalic allodynia (*p* = 0.03), than those with EM. Concurrent medical disease were denoted in 58 patients with migraine; endocrinologic (*n* = 13), cardiovascular (*n* = 12), gastrointestinal (*n* = 9), neurological (*n* = 7), orthopedical (*n* = 7), otolaryngeal (*n* = 7), pulmonary (n = 3), hematologic (*n* = 2), ophthalmologic (*n* = 2), and dermatologic diseases (*n* = 1). Its frequency between EM and CM was not different.

**Table 1:** Demographic and clinical characteristics with respect to headache chronicity

	**Mean ± SD (range) or number (%)**		***p *** value*
	**Episodic migraine**	**Chronic migraine**	
	**(*n*** **= 183)**	**(*n*** **= 68)**	
**Age, year**	38.0 ± 12.7 (15-65)	41.4 ± 13.4 (15-70)	0.063
**Gender, female**	152 (83.1)	60 (88.2)	0.433
**Education, year**	13.0 ± 3.2 (6-18)	11.5 ± 3.5 (4-18)	0.002
**Job, yes**	81 (44.3)	27 (39.7)	0.568
**Income, at least 1 million KRW/month**	73 (39.9)	24 (35.3)	0.561
**Marriage, married but not divorced or bereaved**	114 (62.3)	50 (73.5)	0.103
**Concurrent medical disease, yes**	43 (23.5)	15 (22.1)	0.868
**Age at onset, year**	27.5 ± 11.4 (7-55)	29.0 ± 12.7 (6-63)	0.341
**Disease duration, year**	10.5 ± 8.9 (1-40)	12.4 ± 9.3 (1-40)	0.138
**Attack frequency, /3 months**	7.2 ± 8.3 (1-50)	28.2 ± 26.6 (5-90)	<0.001
**Attack duration, hour**	26.4 ± 25.9 (2-72)	32.7 ± 32.9 (3-120)	0.112
**VAS**_**max**_^**†**^	7.6 ± 2.3 (0-10)	8.2 ± 1.7 (3-10)	0.106
**VAS**_**now**_^**‡**^	2.1 ± 2.2 (0-10)	3.9 ± 2.7 (0-10)	<0.001
**Photophobia**	78 (42.6)	37 (54.4)	0.117
**Phonophobia**	104 (56.8)	47 (69.1)	0.083
**Cephalic allodynia**	22 (12.0)	16 (23.5)	0.03
**Family history**	105 (57.4)	38 (55.9)	0.886

^*^Independent *t* test, Mann-Whitney *U* test, or Fisher’s exact test was applied.

^†^Maximal headache intensity during the preceding 3 months.

^‡^Headache intensity on the day conducting the questionnaires.

KRW: Korean Won, VAS: Visual Analog Scale.

Migraine disability, psychiatric symptoms, and QOL comparisons between EM and CM are listed in Table 2. Patients with CM showed a higher mean MIDAS score than those with EM (*p* < 0.001), and the proportion of patients with migraine with MIDAS score ≥ 21 (grade IV severe disability) was also higher for CM, at 70.6% versus 37.2% for EM (*p* < 0.001). Patients with CM had a higher mean BDI score than those with EM (*p* < 0.001), as well as a higher frequency of depression (*p* = 0.001). The results of the mean BAI score and the frequency of anxiety were comparable to those of the mean BDI score and the frequency of depression. Finally, the mean MSQoL score was lower in patients with CM than those with EM (*p* < 0.001).

**Table 2:** Migraine-associated disability, psychiatric symptoms, and quality of life with respect to headache chronicity

	**Mean ± SD (range) or number (%)**		***p *** value*
	**Episodic migraine**	**Chronic migraine**	
	**(*n*** **= 183)**	**(*n*** **= 68)**	
**MIDAS score**	26.5 ± 32.6 (0-180)	54.1 ± 49.9 (0-180)	<0.001
**MIDAS, grade III and IV**	114 (62.3)	55 (80.9)	0.006
**BDI score**	13.4 ± 9.7 (0-42)	18.7 ± 10.5 (0-46)	<0.001
**Depression, >16 on BDI**	55 (30.1)	36 (52.9)	0.001
**BAI score**	12.6 ± 10.0 (0-47)	18.9 ± 12.6 (1-61)	<0.001
**Anxiety, >21 on BAI**	32 (17.5)	26 (38.2)	0.001
**MSQoL score**	67.3 ± 15.0 (29-96)	56.0 ± 16.5 (25-94)	<0.001

^*^Independent *t* test, Mann-Whitney *U* test, or Fisher’s exact test was applied.

MIDAS: Migraine Disability Assessment Scale, BDI: Beck Depression Inventory, BAI: Beck Anxiety Inventory, MSQoL: Migraine-Specific Quality of Life.

Predictors of the MSQoL score determined by stepwise linear regression analyses are depicted in Table 3. The strongest predictor was the BDI score (β = -0.373, *p* < 0.001), followed by the MIDAS score (β = -0.223, *p* < 0.001), gender (β = -0.192, *p* < 0.001), attack duration (β = -0.159, *p* = 0.001) and headache chronicity (β = -0.130, *p* = 0.012). In other words, high depression symptoms, high migraine disability, female gender, a long duration of headache, and CM all contributed to decreasing QOL. Stepwise regression produced a five-variable model that explained 42.7% of the variance in the MSQoL score. According to the standardized β, the contribution of BDI score to QOL was 1.67 times higher than that of the MIDAS score, 1.94 times higher than that of gender, 2.35 times higher than that of attack duration, and 2.87 times higher than that of headache chronicity. Colinearity statistics indicated that these variables had independent effects without redundancy.

**Table 3:** Predictors determining the MSQoL score by stepwise multiple linear regression analysis

**Variable**	**Standardized coefficient β**	***p *** **value**	**Collinearity (VIF)**	**Adjusted *R***^***2***^
				0.427
**BDI score**	−0.373	<0.001	1.206	
**MIDAS score**	−0.223	<0.001	1.238	
**Gender**	−0.192	<0.001	1.047	
**Attack duration**	−0.159	0.001	1.031	
**Headache chronicity**	−0.130	0.012	1.131	

MSQoL: Migraine-Specific Quality of Life, BDI: Beck Depression Inventory, MIDAS: Migraine Disability Assessment Scale.

**Figure 1:**
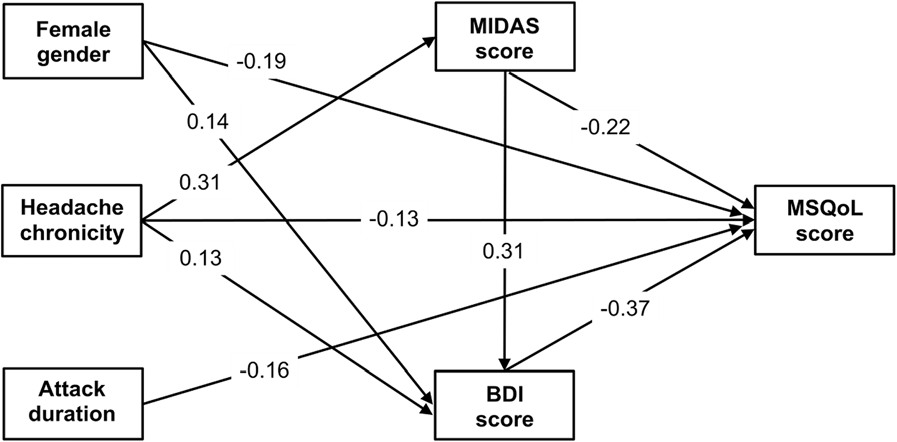
**Interrelationships between clinical variables and Migraine-Specific Quality of Life (MSQoL) score by refined path analysis model.** An arrow indicates a direct relationship from variable to another. Numbers denote standardized regression coefficients (beta weights) for each path. If the sign of the coefficient is negative, when the predictor variable score increases by 1 standard deviation, MSQoL score decreases by the number of standard deviations as indicated by the value of the coefficient. All regression coefficients are statistically significant (*p*<0.01). MIDAS: Migraine Disability Assessment Scale, BDI: Beck Depression Inventory, MSQoL: Migraine-Specific Quality of Life.

Complex interrelationships between predictors and the MSQoL score were illustrated by the refined path analysis model in Figure 1. According to pre-defined criteria, the final model provided an excellent fit to the data (χ^*2*^ = 3.12, *p* = 0.37; NFI = 0.99; CFI = 1; GFI = 1; RMR = 0.025). All regression coefficients were statistically significant (*p* < 0.01). Gender, headache chronicity, attack duration, the MIDAS score, and the BDI score exerted direct effects on the MSQoL score. Female gender exerted an indirect effect on the MSQoL via the BDI score. Headache chronicity revealed an indirect effect on the MSQoL score through the MIDAS and the BDI scores. The MIDAS score exerted an indirect effect on the MSQoL through the BDI score. Unlikely to the hypothesized path model, gender did not have an indirect effect on the MIDAS score and attack duration did not exert an indirect effect on the MIDAS and BDI scores.

## Discussion

We determined that almost one-fourth of eligible patients had CM. Patients with CM were more likely to have a low education level, a high attack frequency, a large pain intensity, and a high frequency of cephalic allodynia than those with EM. Furthermore, they appeared to have greater migraine disability, higher depression and anxiety symptoms, and poorer QOL than those with EM. Among these variables, depression symptoms, migraine-associated disability, being female, long duration of headaches, and headache chronicity mainly contributed to the decreased QOL. Headache chronicity had a direct effect on QOL and indirect effects on QOL, inducing migraine disability and depression.

The prevalence of CM with respect to the study design is different. Only about 5–6% of respondents were diagnosed with CM in the larger population-based studies [12,14], whereas more than half of patients with migraine demonstrated CM in several hospital-based studies [31,32]. The frequency of CM in our patients was within the range of the hospital-based studies. The features of clinical and demographic characteristics of patients with CM in this study were similar to those of patients in other studies [13,14,33,34]. In general, the frequency of cutaneous allodynia in patients with migraine is about 60–70%, and does not differ between CM and EM [34]. Contrary to this finding, in our study the frequency of cephalic allodynia was somewhat lower, by as much as 22% in patients with CM and 16% in those with EM, and patients with CM demonstrated a significantly higher frequency of it than those with EM. The low frequency of allodynia in our study may be explained by three ways. First, we measured only cephalic allodynia, ignoring extracephalic allodynia originated from body or extremities. Second, we did not examine allodynia, by using a specific tool such as quantitative sensory testing or dynamic mechanical method including brushing. Third, the low frequency of allodynia may be related to the ethnic difference. A further study to compare the prevalence of allodynia in one race versus another should be done.

Headache chronicity has been related to headache disability, emotional burden and QOL in two large observational studies (the AMPP and IBMS studies) and one hospital-based Taiwanese study [11-14,32]. The AMPP study is a large, United States (US) population-based longitudinal survey that identified 24,000 respondents with headache and followed them annually for 5 years [11,14]. The IBMS is a web-based, cross-sectional, multinational survey that identified and evaluated persons with CM or EM [12,13]. The Taiwanese study is a well-designed, clinic-based study, which recruited as many patients with CM as with EM (CM, n = 167 (50.5%) vs. EM, n = 164 (49.5%)) [32]. In the IBMS study, the frequency of MIDAS grade IV scores was 78% for CM and 23.3% for EM [12], and in the Taiwanese study, 59.3% for CM and 21.9% for EM [32]. Our study also demonstrated a greater headache-related disability in patients with CM than those with EM. The frequency of MIDAS grade IV scores was 70.6% in patients with CM, which was almost double that of patients with EM (35.2%). We further found that the frequency of depression in patients with CM was 52.9%, which was higher than that of those with EM (30.1%). The frequency of anxiety was also higher in patients with CM (38.2%) than with EM (17.5%). Consistent with our study, the levels of anxiety and depression were higher in patients with CM in both population-based and hospital-based studies. The AMPP study found that patients with CM were twice as likely to have depression and anxiety compared with those with EM [14]. The IBMS study and the Taiwanese study also reported that moderate to severe anxiety and depression were specifically more prominent for CM compared to EM (47.0% vs. 25.1% in IBMS, 33.5% vs. 7.9% in the Taiwanese study) [13,32].

One benefit of our study is clarification of the interrelationships among the variables that influence QOL. We found that headache chronicity had both direct and indirect effects on QOL by inducing migraine disability and depression. Headache chronicity has a weak direct relationship to MSQoL when compared with its indirect relationships. We suggest that the impact of headache chronicity on QOL might be stronger when it induces disability and depression. An interesting point in our study was that female gender was one of the contributing factors in determining QOL directly, as well as indirectly by provoking depression. Several representative population-based studies examining the disease burden of migraine did not identify female gender as a risk factor for QOL [19,20,24]. Only one clinic-based study suggested female gender was a significant predictor of impaired QOL [22]. The pathophysiological mechanism between female gender and QOL in migraine is not clear, but certain reproduction-related hormonal changes might place women at increased risk for depression, migraine and increased chronicity of headache [35,36]. These notable gender differences could bring about impaired QOL, but to confirm this link, further study about gender-specific influences of migraine on QOL would be required.

## Conclusion

In this study, we demonstrated that headache chronicity and its impact on headache disability also elicited depression. Therefore, early identification and appropriate management of CM might lessen headache disability and depression both, and thereby improve QOL. Recent trials of topiramate and botulinum toxin type A were also encouraging, demonstrating that the drugs improved QOL in patients with CM [37]. Another way to improve QOL would be to screen for depression symptoms when the patients visit a headache clinic. Brief, self-reported questionnaires such as the Hospital Anxiety and Depression Scale (HADS) or the 9-item Patient Health Questionnaire (PHQ-9) may be enough to detect symptoms early [38]. Although no studies have proven that the treatment of depression in patients with CM would improve their QOL, our study can act as a springboard towards future investigation of that issue.

Some limitations should be considered when interpreting the results of this study. First, this is a hospital-based study, so seriously ill individuals were more likely to be recruited, and thus their comorbidities might be overestimated. Therefore, a population-based study to measure QOL is needed. Second, we used only self-administered psychometric instruments to test our eligible patients. This protocol did not include structured interviews, such as the Structured Clinical Interview for DSM-IV axis I disorders (SCID) [39], which are considered the gold standard for research regarding psychiatric problems. However, the BDI and BAI are good screening tools for depression and anxiety for physicians other than psychiatrists, and are both well-validated tests frequently used for screening and for grading in headache studies [40]. Third, we did not use the MSQ 2.1, which is the more popular way to measure QOL in patients with EM and CM [41]. The MSQ 2.1 is the most frequently utilized tool assessing migraine-specific QOL, but its reliability and validity have not yet been measured in Korean patients with migraine. Future studies to identify predictors of QOL using the MSQ 2.1 in a Korean population will need to be done.

In summary, patients with CM had more serious clinical, psychiatric, and poor QOL than did patients with EM. CM appears to impair QOL directly as well as indirectly by provoking disability and depression.

## Competing interests

The authors have no financial conflicts of interests.

## Authors’ contributions

SP Park took part in the design of the study, contributed to the data collection. SY Kim participated in writing the manuscript. Both authors agreed to accept equal responsibility for the accuracy of the content of the paper. Both authors read and approved the final manuscript.
